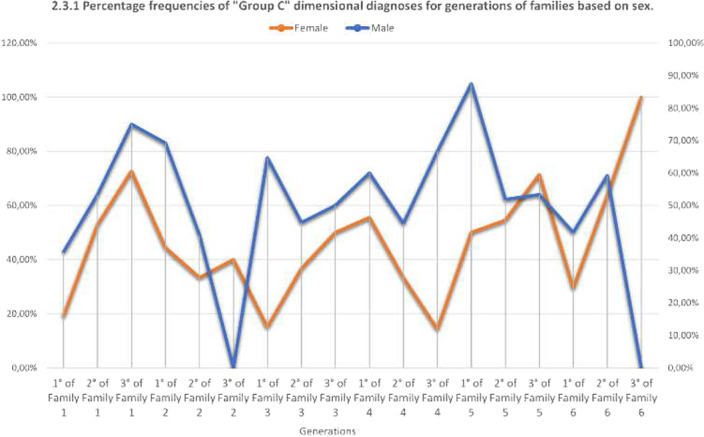# Local and exhaustive study of transmission of detected psychopathological dimensions in family groups in the “albanova” area

**DOI:** 10.1192/j.eurpsy.2021.858

**Published:** 2021-08-13

**Authors:** C. Serritella, A. Russo, G. Rossi, M. Paolino, M. Baldascino, H. Del Vecchio, M. Cimmino, T. Salvati

**Affiliations:** 1 Department Of Mental Health (dsm), Mental Health Operational Unit (Uosm) Of San Cipriano D’aversa, Caserta, Italy; 2 Department Of Economics, University Of Studies Of Campania Luigi Vanvitelli, Caserta, Italy; 3 Cnr Napoli, Genetics and biophysics “Adriano Buzzati Traverso”, Caserta, Italy; 4 Chair Of Evolutionary Age, University Of Studies Of Naples Suor Orsola Benincasa, Caserta, Italy

**Keywords:** Phenotipe, genetics, epigenetics, Psychopatological

## Abstract

**Introduction:**

It is not known with certainty how different phenotypes are transmitted in groups of families divided into three generations.

**Objectives:**

Having meticulously searched for terms of psychopathogic lexicon that best translated the sterile categorical diagnosis, we obtained three dimensional groups for all six families in the three generations.

**Methods:**

We calculated the frequencies and percentages of the three dimensional groups for the three generations of families based on sex.

**Results:**

The chi-square TEST attests a p-value = 0.049, statistically significant for the dimensional group “A”. (Tab. 3)

**Figure:**
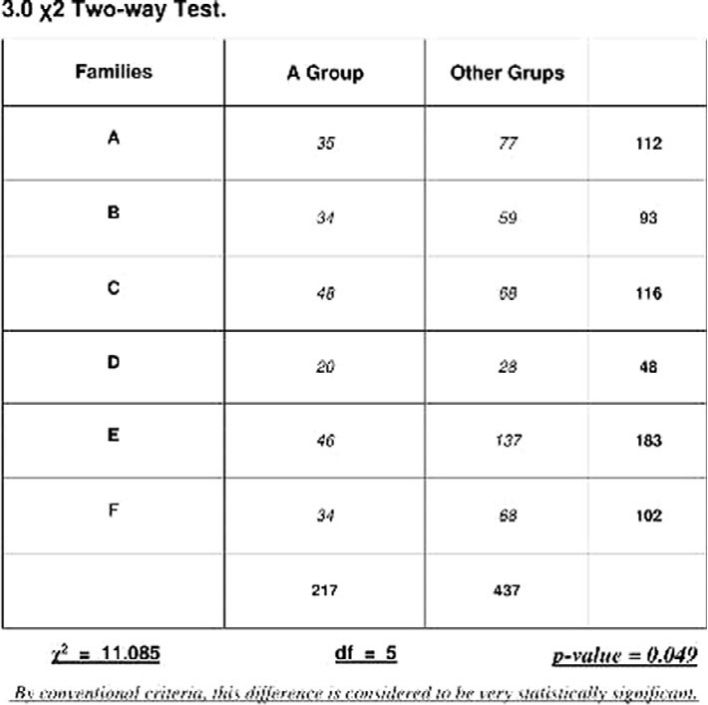

**Conclusions:**

The genetics, and above all the epigenetics, of the phenotypes are periodically transmitted in group “A” and group “C” in the female and male sex. (Graphs 2.1.1 and 2.3.1) Different phenotypes indicate that the complexity of the interactions of the regulatory mechanisms of genes with the environment is extremely significant for the group with the most severe psychiatric pathology.

**Figure:**
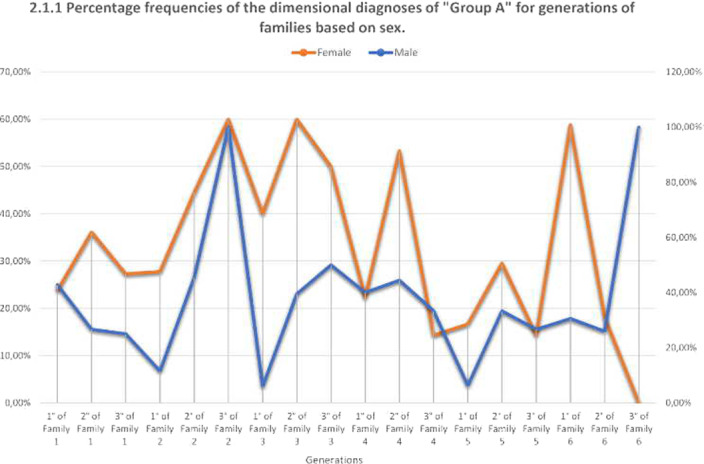

**Figure:**